# Editorial: The Role of Declarative and Procedural Memory in Language

**DOI:** 10.3389/fpsyg.2021.816889

**Published:** 2021-12-22

**Authors:** Nicolas Stefaniak, Stéphanie Caillies, Ferenc Kemény

**Affiliations:** ^1^Laboratoire C2S (Cognition, Santé, Société), Université de Reims Champagne-Ardenne, Reims, France; ^2^Institute of Psychology, University of Graz, Graz, Austria; ^3^Institute of Education and Psychology at Szombathely, Eötvös Loránd University, Budapest, Hungary

**Keywords:** Declarative/Procedural model, statistical learning, language, declarative memory, procedural memory, thalamus, specific language impairment (SLI), developmental learning disorder

It is becoming increasingly clear that language at least partially depends on learning and memory processes, which have been conceptualized somewhat differently according to areas of research. Automatic processes have often been thought of in terms of statistical learning, implicit learning, or procedural learning and memory systems. In contrast, controlled processes should be supported by explicit knowledge or declarative learning and memory systems. Typical language comprehension and production seem to involve both types of memory and processes, which interact during efficient communication.

The Declarative/Procedural model proposed by Ullman (2001) represents one of the most influential models to account for the role of memory systems in language in the last 20 years. According to this model, procedural memory would be involved in the manipulation of grammar, including syntax, morphology, and phonology while declarative memory would sustain the mental lexicon, meaning, word sounds, and the acquisition of irregular grammatical rules. Based on this model, hypotheses about the origin of language impairment emerged. Indeed, specific language impairment (Ullman and Pierpont, 2005) and other developmental disorders of language (Ullman et al., 2020) could be explained by an impairment in procedural memory. This hypothesis is known as the procedural deficit hypothesis.

This Research Topic helps to bring a more fine-grained vision of the role of both memory systems by attributing functions that were not clearly devoted to one memory system (Arthur et al.) or by attributing new functions to procedural memory (Crosson; Kemeny and Lukács; Stefaniak et al.). More specifically, it appears that phonological processing would depend more on declarative memory instead of procedural memory (Arthur et al.), while procedural memory could also be more involved in the acquisition of word lexicon (Kemeny and Lukács), as well as the selection of words while inhibiting concurrent words (Crosson), which could support the process to find meaning (Stefaniak et al.). Llompart and Dabrowska go even further to challenge the Declarative/Procedural model and suggest that grammatical and lexical knowledge depends on the same cognitive mechanisms. Altogether, these studies highlight that both memory systems are likely more intertwined in each aspect of language than initially thought and that more dynamical interactions between both memory systems are required (Stefaniak et al.). This role could be devoted to the thalamus (Crosson). Interestingly, although studies in our Research Topic help to better characterize the role of both memory systems in language, it does not challenge the procedural deficit hypothesis. Indeed, Kemeny and Lukács showed that the link between lexical acquisition and procedural memory is only observed in typically developing children, but that lexical acquisition depends more on verbal short-term memory in children with language impairment, which is not inconsistent with the procedural deficit hypothesis. Moreover, Hollander and Adi-Japha showed that developmental disorders of both oral and written language were deeply related to motor impairment, which supports the procedural deficit hypothesis. Nevertheless, Hollander and Adi-Japha also remind that the educational environment must not be underestimated in the occurrence of developmental learning disorders.

Altogether, this Research Topic opens new directions for future research. First, a more in-depth analysis should be made to determine the commonalities and the differences between the Declarative/Procedural model and the statistical learning framework. Second, this Research Topic highlights that it is still far from being clear to precisely state the role of both memory systems. Further work is required to specify the functions of the different memory systems in language—both in children and in adults. Third, future research must also explore how the new roles devoted to the procedural and declarative memory impact developmental learning impairment.

Finally, editors of this Research Topic wish to warmly thank authors who took part to this Research Topic.

## Author Contributions

All authors listed have made a substantial, direct, and intellectual contribution to the work and approved it for publication.

## Conflict of Interest

The authors declare that the research was conducted in the absence of any commercial or financial relationships that could be construed as a potential conflict of interest.

## Publisher's Note

All claims expressed in this article are solely those of the authors and do not necessarily represent those of their affiliated organizations, or those of the publisher, the editors and the reviewers. Any product that may be evaluated in this article, or claim that may be made by its manufacturer, is not guaranteed or endorsed by the publisher.
